# Stromal Expression Profiling Reveals Immune‐Driven Adaption to Malignancy in Canine Melanoma Subtypes

**DOI:** 10.1111/vco.13021

**Published:** 2024-10-17

**Authors:** Erin Beebe, Christiane Krudewig, Zahra Motamed, Alexandra Malbon, Enni Markkanen

**Affiliations:** ^1^ Institute of Veterinary Pharmacology and Toxicology, Vetsuisse Faculty University of Zurich Zurich Switzerland; ^2^ Institute of Veterinary Pathology, Vetsuisse Faculty University of Zurich Zurich Switzerland; ^3^ The Royal (Dick) School of Veterinary Studies and the Roslin Institute Midlothian UK

**Keywords:** cancer‐associated stroma, canine melanoma, comparative oncology, laser‐capture microdissection, RNA sequencing

## Abstract

Canine mucosal melanoma (CMM) is the most common oral malignancy in dogs and is significantly more aggressive than its cutaneous counterpart (CCM), yet the reasons for this disparity remain unclear. Cancer‐associated stroma (CAS) plays a crucial role in tumour progression, but a detailed understanding of CAS in canine melanoma is missing. To assess stromal reprogramming, we analysed CAS from 21 CMM, 14 CCM and normal stroma from 10 skin and 9 oral mucosa samples by laser‐capture microdissection followed by RNA sequencing. Results were assessed in relation to subtypes, prognostic factors including mitotic count (MC), ulceration, necrosis, pigmentation and immune cell infiltration (CD3, CD20 and CD68), scored using immunohistochemistry and RNA in situ hybridisation. Stromal reprogramming was evident in both subtypes but significantly more pronounced in CMM. Immune‐excluded tumours exhibited higher MC than desert/cold ones. MC strongly correlated with genes associated with B‐cells, T‐helper cells and CTLA4 in CCM, suggesting CAS reprogramming to depend on tumour malignancy. Finally, we identify an immune‐suppressive stromal signature in a subset of CMM characterised by the downregulation of key immune checkpoints and pathways. Together, these findings provide a solid foundation for understanding the role of CAS in canine melanoma, specific to cutaneous and mucosal subtypes.

## 1 Introduction

Melanoma is the most common oral malignancy in dogs but can also affect cutaneous sites. While only a minor subset of canine cutaneous melanocytic (CCM) tumours show malignant behaviour, canine mucosal melanocytic (CMM) tumours are often highly aggressive, frequently resulting in metastatic disease and poor prognosis, yet reasons behind this stark difference in risk of disease progression are poorly understood [1]. Adverse prognostic indicators include primary location, tumour ulceration or necrosis, amelanotic tumours and age at the time of diagnosis [2, 3, 4]. Tumour mitotic count (MC) remains the most important histological criterium for diagnosis and prediction of patient outcomes [5]. Importantly however, existing prognostic markers focus solely on tumour‐based characterisation, while the contributing role and coordination of the cancer‐associated stroma (CAS) remains ambiguous, especially in the presence of different degrees of malignancy, as observed in CCM and CMM.

CAS is increasingly recognised for facilitating tumour dissemination and local invasion, also in human melanoma [6]. Furthermore, CAS profiling according to tumour‐infiltrating lymphocyte density supports patient stratification for immunotherapy in human patients [7, 8]. Aside from immune cells, cancer‐associated fibroblasts (CAFs) are the dominant cell type in CAS. Activation of CAFs, in the presence of malignant growth, results in the excessive secretion of a cocktail of cytokines and other extracellular matrix (ECM) proteins that triggers a downstream cascade of events linked to disease progression [9]. However, translation of these findings to canine cancer biology is lacking.

Steps to characterise the tumour microenvironment in canine melanoma, according to lymphocyte infiltration, revealed high levels of CD20+ lymphocytes associated with both clinical outcome and the presence of metastasis [10]. A follow‐up study by the same group also revealed a link between increased CD163+ macrophages and metastases in canines with oral melanoma [11]. Furthermore, expression of certain checkpoint molecules such as PD‐1 and PD‐L1 was shown to correlate with increased CD3+ T‐cells [12]. As such, the use of immunotherapy in dogs is expected to become a key addition to future therapeutics. Unfortunately, current literature is limited by the lack of detailed investigation into other components of CAS, with one sequencing study performed on whole tissue sections from four melanocytomas and two melanomas, while other larger scale studies have focussed on the mapping of tumour‐specific mutations, making it difficult to stratify patients for therapy and predict patient response [13, 14, 15, 16].

To investigate CAS‐specific reprogramming in CCM and CMM in molecular detail, we applied laser‐capture microdissection (LCM) coupled with RNA sequencing, as previously established in the lab [17, 18, 19, 20, 21, 22]. Using this approach, we analysed a cohort of 14 CCM, 21 CMM, 10 cutaneous normal (cNormal) and 9 mucosal normal (mNormal) tissue samples. This was supported by histological examination of prognostic factors and immune infiltration. In doing so, we aimed to highlight stromal expression patterns contributing to tumour malignancy.

## 2 Material and Methods

### 2.1 Cohort Selection

Archival clinical FFPE materials were assessed for stromal content by certified veterinary pathologists (AM & CK), resulting in the selection of 14 cutaneous melanoma (cCAS), 10 cNormal, 21 oral mucosal melanoma (mCAS) and 9 mNormal cases suitable for LCM (Table S1, Figure S1).

Cell line validation statement: No cell lines used.

### 2.2 LCM and RNA Isolation

Slide preparation was performed as previously published [23, 24]. In brief, 10 μm sections of FFPE tissue were mounted on PEN membrane glass slides (Applied Biosystems). LCM was performed using the ArcturusXT system (Thermo Scientific) together with CapSure Macro LCM Caps (Applied Biosystems), followed by visual confirmation of successful isolation (Figure S2). RNA was isolated using the Covaris truXTRAC FFPE RNA kit. Quality and concentration of the eluted RNA were measured using the Agilent 4200 TapeStation System and High Sensitivity RNA ScreenTape kit (Agilent Technologies), according to the manufacturer's protocol. RNA integrity number (RIN) ranged from 4.1 to 1.1, while the percentage of RNA fragments greater than 200 (DV200) ranged from 94.95% to 32.13% (Table S1). All samples with sufficient concentration were submitted for sequencing at the Functional Genomics Center Zurich (FGCZ).

### 2.3 RNA Sequencing

10 ng of RNA was submitted for sequencing at the FGCZ. Library preparation was performed with the SMARTer Stranded Total RNA‐Seq Kit—Pico Input Mammalian (Takara Bio) according to the manufacturer's protocol. Sequencing was run on the Illumina Novaseq (single‐end, 100 bp) according to standard protocols used at the FGCZ. The quality of resulting reads was checked with FastQC (Babraham Bioinformatics) before pseudoalignment to the reference transcriptome (CanFam3.1, Ensembl, version 104) and quantification using Kallisto (Version 0.50.0) [25]. Sequencing data has been deposited to the NCBI Gene Expression Omnibus and is available under accession GSE266234.

### 2.4 Bioinformatics Analysis and Data Visualisation

Kallisto abundance files were read into RStudio and summarised at the gene level using Tximport and biomaRt [26, 27]. Raw counts were filtered considering at least 10 counts according to the smallest biological group per comparison. Principle component analysis (PCA) was performed on VST normalised counts and visualised using ggplot2 [28]. Bioconductor package DESeq2 was used to perform differential gene expression analysis, and all significant differentially expressed genes (DEGs) were identified according to padj < 0.05 and log2FC > |1| [29].

Volcano plots depicting significant DEGs were generated using ggplot2 [28]. CCM pathway analysis was performed with MsigDB webtool using compute overlap with selection for all canonical pathways [30, 31]. Alternatively, gene set enrichment analysis (GSEA) for CMM was performed in WebGestalt using reactome pathways [32]. Heatmap visualisation of DEGs and clustering was performed using ComplexHeatmap with HeatmapAnnotation set according to group and Euclidean row clustering [33]. Pearson correlation coefficients and significance between MC and stromal expression were calculated and visualised using package ggcorrplot [34]. Unless otherwise stated, all other plots and statistical testing were performed using GraphPad Prism (version 10.2.2).

### 2.5 Validation

Validation of RNAseq results by RT‐qPCR was performed using 10 ng of LCM‐FFPE RNA, as previously described [19, 23]. Reverse transcription using iScript cDNA Synthesis Kit (BioRad) was followed by TaqMan Preamplification and qPCR. A table of taqman primers can be found in Table S2. Relative mRNA expression was quantified using the ddCt method, taking the mean of PPIA and B2M as housekeeping genes.

### 2.6 Histological Scoring of Prognostic Factors and Immune Infiltration

All histological scoring was performed by veterinary pathologist (CK). Tumour cases were retrospectively scored for different prognostic indicators after sequencing. The presence of ulceration, necrosis and degree of tumour pigmentation were performed using standard H&E slides and scored in line with a previous publication [10]. MC was assessed on H&E stained slides by counting mitotic numbers in 10 consecutive high power fields (total 2.37 mm^2^) of tumour tissue.

Tumour‐infiltrating immune cells were detected using in situ hybridisation RNAscope 2.5 HD Assay according to protocol guidelines (ACD probes: CD3+, #406271; and CD68+, #577861; negative control DapB, #310043; positive control polr2a, #310981). Additional detection of CD20+ immune cell was performed using immunohistochemistry (#RB‐9013‐P, Epredia, 1:200) with lymph node tissue as a positive control. All stained slides were scanned (C10730 series NanoZoomer‐RS/NanoZoomer‐HT C9600 series) and visualised using Visiopharm. Semi‐quantitative analysis of tumour infiltration was determined according to a 4‐tier grading scheme as previously published and summarised as either desert/cold (absent or mild density of immune cells regardless of localisation), excluded (moderate or severe density localised at the tumour border) or hot (moderate or severe density with distribution throughout the tumour) [10, 35].

## 3 Results

### 3.1 Transcriptomic Characterisation Reveals Limited Stromal Reprogramming in Canine Cutaneous and Mucosal Melanoma Subtypes

**FIGURE 1:**
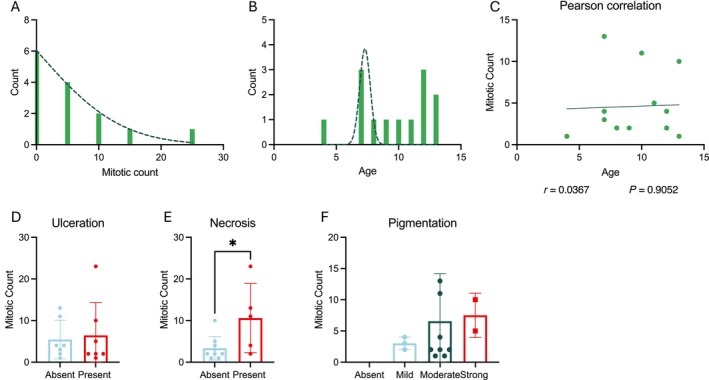
Characterisation of CCM cohort according to common prognostic factors. (A) Frequency distribution of tumour mitotic count. (B) Frequency distribution of patient age. (C) Pearson correlation of patient age with tumour mitotic count. (D) Association between ulceration and mitotic count. (E) Association between necrosis and mitotic count. *p* values calculated using unpaired *t*‐test. (F) Association between pigmentation and mitotic count. Key: *p* < 0.05 = *.

Initial characterisation of our CCM cohort according to commonly documented prognostic factors revealed that 12/14 cases have a MC below or equal to 10 (Figure 1A), and age was distributed randomly between 4 and 13 years old (median age: 7 years, Figure 1B). No correlation was observed between MC and age (Figure 1C). Furthermore, our cutaneous cohort was comprised of 7/14 cases present for ulceration and 5/14 cases present for necrosis. Meanwhile, 3, 9 and 2 cases presented with mild, moderate and strong pigmentation, respectively (Table S1). Lastly, no association between MC and ulceration or pigmentation was observed (Figure 1D,F). Instead, the presence of necrosis was associated with significantly higher MC in CCM (Figure 1E).

**FIGURE 2:**
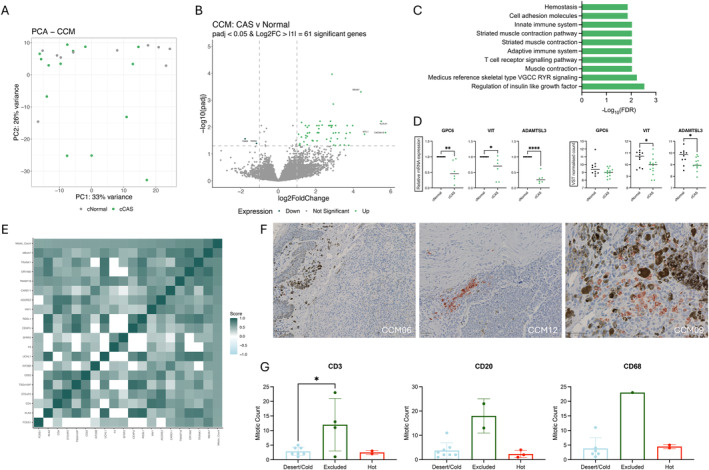
Characterisation of stromal reprogramming in CCM using RNA sequencing. (A) PCA of VST normalised counts in CCM CAS and normal stroma. cNormal in grey and cCAS in green. (B) Volcano plot highlighting significant differentially expressed genes in CCM CAS compared to normal using padj < 0.05 and Log2FC > |1| as cut‐off values. (C) Over‐representation analysis of canonical pathways in significantly upregulated genes in CCM using MSigDB. (D) Validation of selected genes from CCM data using RT‐qPCR. *p* values calculated using unpaired *t*‐test. (E) Correlation matrix of DEG expression with tumour mitotic count using *p* < 0.05 and *r* > |0.5| as cut‐off values. (F) Representative images of CD20 IHC for desert/cold, excluded and hot infiltration in CCM cases. (G) Association between CCM tumour immune infiltration and mitotic count analysed using one‐way ANOVA. Key: *p* < 0.05 = *, *p* < 0.01 = **, *p* < 0.001 = ***, *p* < 0.0001 = ****.

Next, principal component analysis (PCA) was applied to project key differences in stromal gene expression profiles in cutaneous CAS (cCAS) compared to cNormal and evaluate sample clustering within this melanoma subtype. In doing so, we observed a substantial overlap of CAS in CCM with the respective normal samples (Figure 2A). Furthermore, DGEA identified 61 significant genes within significance thresholds of padj < 0.05 and Log2FC > |1| (Figure 2B, Table S3). Among these, only two genes were significantly downregulated in cCAS, namely TGM3 and RORA. Validation of sequencing results for select DEGs in CCM was performed using RT‐qPCR (Figure 2C). Upregulated genes were enriched for pathways including insulin‐like growth factor regulation, muscle contraction and several immune‐associated pathways, including T‐cell receptor signalling, adaptive and innate immune system, driven by increased expression of CD4, CARD11, CTLA4 and CD22 among others (Figure 2D).

Subsequent analysis identified expression of 19/61 DEGs to strongly correlate with tumour MC (*p* < 0.05 and correlation coefficient > |0.5|, Figure 2E, Table S4). Pathway analysis revealed three targets associated with B cell receptor signalling, namely VAV1, CD22 and CARD11.

Further characterisation of tumours according to immune cell infiltration was performed using individual tumour sections stained for CD3 (T‐cell), CD20 (B‐cell) and CD68 (macrophage) that were scored based on degrees of infiltration, as previously described [10, 35]. This score can be summarised as ‘hot’ (immune cells infiltrating tumour tissue), excluded (immune cells present at the tumour periphery), cold (few infiltrating immune cells) and desert (no immune cells detectable), where desert and cold can be viewed as a single group. Staining was visualised using the Visiopharm software and converted to hotspot gradient image for semi‐quantitative interpretation (Figure 2F, Figure S5). An overview of the immune infiltration score per case can be found in Table S1. As such, the analysis revealed CD3+, CD20+ and CD68+ excluded tumours to have an increased MC compared to tumours with no immune cell presence (desert/cold) or with high levels of infiltration (hot) (Figure 2G), however should be interpreted with caution due to low sample size.

### 3.2 Limited Interdependence Between Classical Prognostic Factors and Stromal Reprogramming in CMM

**FIGURE 3:**
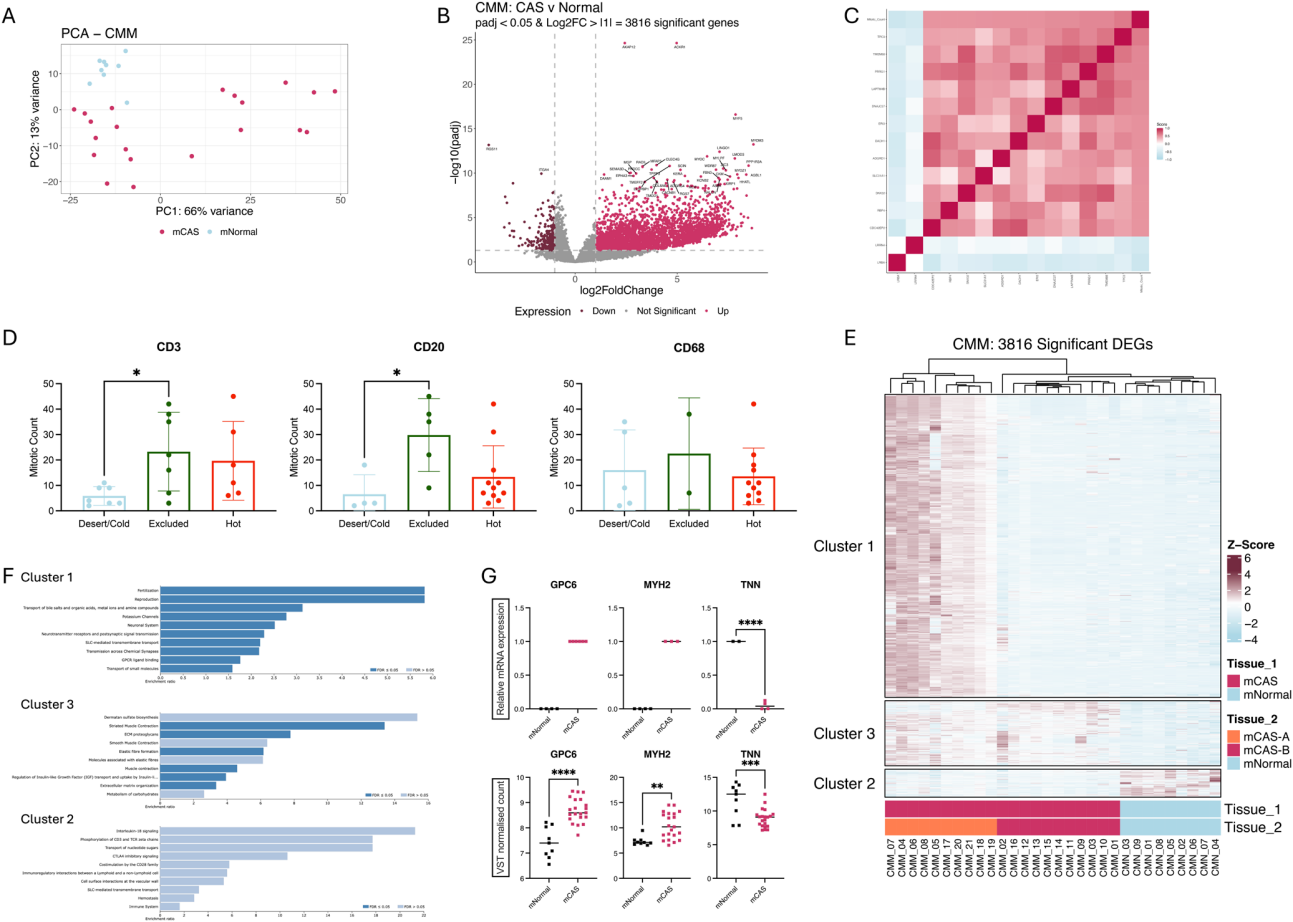
Characterisation of stromal reprogramming in CMM using RNA sequencing. **(**A) PCA of VST normalised counts in CMM CAS and normal stroma. mNormal in light blue and mCAS in dark pink. (B) Volcano plot highlighting significant differentially expressed genes in CMM CAS compared to normal using padj < 0.05 and Log2FC > |1| as cut‐off values. (C) Correlation matrix of stromal DEG expression with tumour mitotic count using *p* < 0.05 and *r* > |0.5| as cut‐off values. (D) Association between CMM tumour immune infiltration and mitotic count analysed using one‐way ANOVA. (E) Heatmap of DEG expression in mCAS compared to mNormal. (F) GSEA of Reactome pathways in significantly deregulated genes in CMM according to heatmap clusters using WebGestalt. (G) Validation of selected genes from CMM data using RT‐qPCR. *p* values calculated using unpaired *t*‐test. Key: *p* < 0.05 = *, *p* < 0.01 = **, *p* < 0.001 = ***, *p* < 0.0001 = ****.

Stromal regions isolated from our mucosal cohort derived from tumours of both oral mucosal cavity (mCAS) and mucocutaneous (mcCAS) origin (Table S1). As DGEA between mCAS and mcCAS did not reveal any significant changes (Figure S3), mCAS and mcCAS were grouped together as ‘mCAS’ and considered a single entity in subsequent analysis. PCA revealed distinct clustering between mCAS and the corresponding mNormal tissue samples (Figure 3A). In line with strong stromal remodelling in CMM, we detected 3816 significant DEGs in mCAS compared to mNormal (Figure 3B, Table S5). Of the significant DEGs, 3790 significantly correlated with MC. Further refinement of this list applied correlation cut‐off values of > 0.5 or < −0.5 to identify 14 genes, including Two pore channel 3 (TPC3) and Transmembrane protein 88 (TMEM88) (Figure 3C, Table S6).

Similar to observations in CCM, tumour immune cell exclusion of CD3 and CD20 positive cells was also associated with increased MC in CMM samples (Figure 3D).

Interestingly, heatmap presentation of gene expression per sample revealed two major subgroups of mCAS (Figure 3E). Group A displayed high expression of genes in cluster 1 including developmental pathways such as fertilisation and reproduction, as well as neuronal system and ion transport (Figure 3F). Meanwhile, consistent with all cases of mCAS was high expression of ECM and contractile‐associated pathways (cluster 3, Figure 3F). Interestingly, various immune and immunoregulatory pathways were enriched among downregulated genes in mCAS compared to mNormal (Cluster 2, Figure 3F). Validation of sequencing results for select DEGs in CCM or CMM was performed using RT‐qPCR (Figure 3G).

**FIGURE 4:**
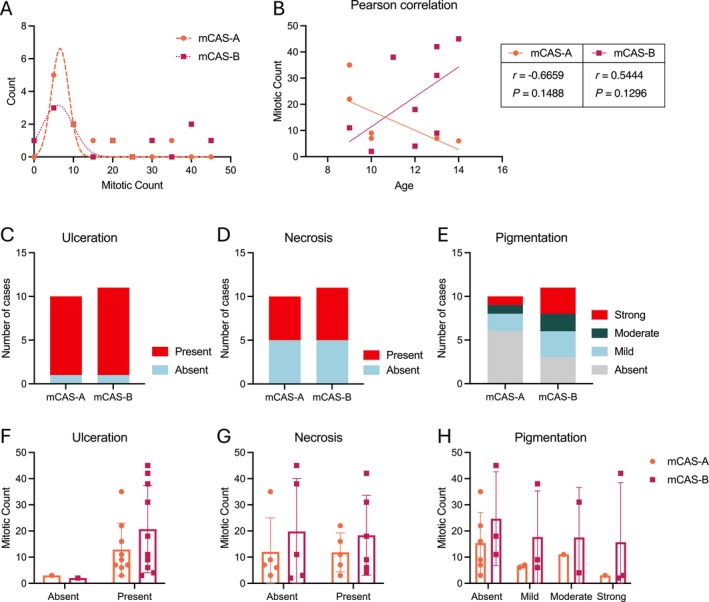
Analysis of common prognostic indicators in CMM tumours according to mCAS subgroup. (A) Frequency distribution of mitotic count in CMM tumours. (B) Pearson correlation of patient age at the time of biopsy in CMM and tumour mitotic count. (C) Occurrence of tumour ulceration. (D) Occurrence of tumour necrosis. (E) Occurrence of tumour pigmentation. (F) Mitotic count according to the absence or presence of ulceration. (G) Mitotic count according to the absence or presence of tumour necrosis. (H) Mitotic count according to tumour pigmentation.

Further characterisation of the two mCAS subgroups in line with common prognostic factors was performed. Frequency distribution analysis of MC showed little difference between mCAS‐A and mCAS‐B (Figure 4A). Furthermore, no significant relationship between age and MC was calculated for either mCAS subgroup (Figure 4B). No further differences were observed in the occurrence of ulceration, necrosis or different degrees of tumour pigmentation (Figure 4C–E). Additionally, no differences were observed in the relationship of prognostic factor with MC (Figure 4F–H).

### 3.3 Identification of CMM Subgroups That Differ With Regards to Immunosuppressive Stromal Features

**FIGURE 5:**
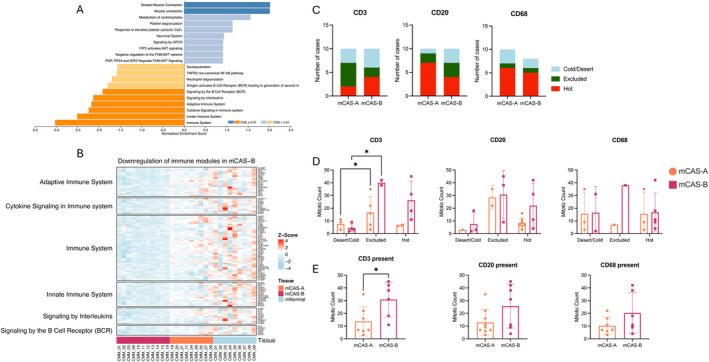
Characterisation of immunosuppression in mCAS subgroups. **(**A) GSEA of reactome pathways for significant DEGs in mCAS‐B compared to mNormal using WebGestalt. (B) Heatmap visualisation of z‐score normalised counts of genes associated with different immune modules as identified from differential gene analysis in mCAS‐B v mNormal. Samples grouped using supervised clustering. (C) Occurrence of CD3+, CD20+ and CD68+ immune cell infiltration into CMM tumours. (D) Evaluation of mitotic count in CMM tumours according to CD3+, CD20+ and CD68+ tumour infiltration. Statistical analysis using 2‐way ANOVA with multiple comparisons. (E) Evaluation of mitotic count in CMM subgroups, mCAS‐A and mCAS‐B according to CD3+, CD20+ and CD68+ tumour infiltration. Statistical analysis using 2‐way ANOVA with multiple comparisons. Key: *p* < 0.05 = *, *p* < 0.01 = **, *p* < 0.001 = ***, *p* < 0.0001 = ****.

Representation of CMM CAS subgroups using a PCA plot revealed clear clustering that could not be explained by primary location (Figure S4AB); however, there was no significant difference in MC between the two subgroups (Figure S4C). Direct comparison of gene expression in mCAS‐A and mCAS‐B compared to mNormal revealed downregulation of key immune‐associated processes in mCAS‐B (Figure 5A), while mCAS‐A was negatively enriched for several RNA processing pathways (Figure S4D). Heatmap presentation of gene expression from the downregulated immune pathways showed the lowest expression in mCAS‐B, while mCAS‐A occupied an intermediate state but was also reduced compared to mNormal stroma (Figure 5B).

We next sought to investigate the role of tumour immune cell recruitment and infiltration in CMM subgroups and its relationship with tumour MC. Overall, the occurrence of CD3, CD20 and CD68 infiltration did not differ significantly between CMM subgroups (Figure 5C). Further assessment revealed significantly higher MC in tumours compared to the desert and cold counterparts (Figure 5D).

Interestingly, MC in the CD3 infiltrated samples was significantly higher in the mCAS‐B group than in the mCAS‐A. The same trend was observed for both CD20 and CD68 infiltration, though these did not quite reach statistical significance (Figure 5E).

**FIGURE 6:**
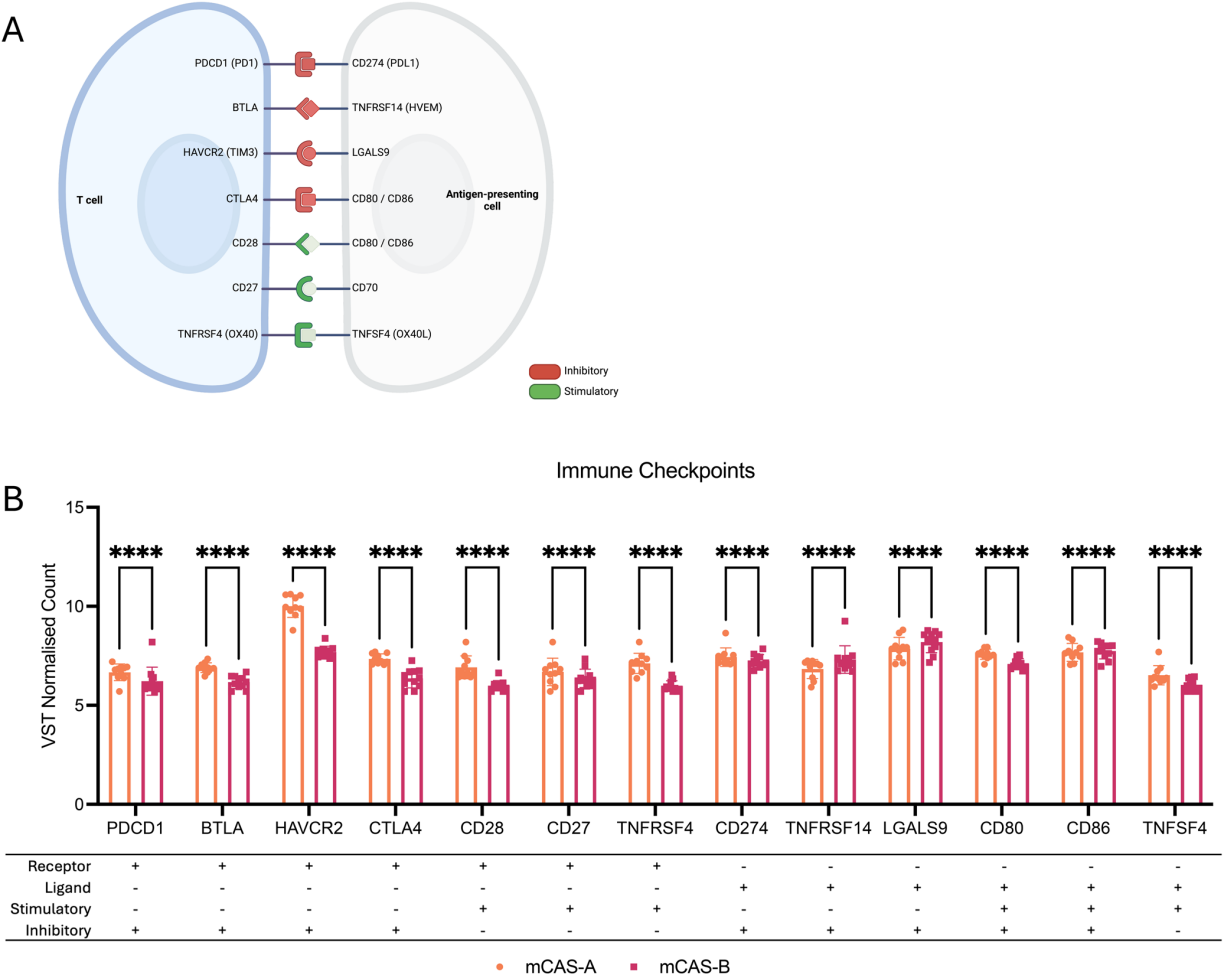
Stromal expression of Immune checkpoints in CMM subgroups. (A) Summary of common immune checkpoints with assumed inhibitory or stimulatory function and associated expression on antigen‐presenting cell or T‐cell. (B) VST normalised counts of immune checkpoints according to mCAS subgroups with table summarising function. Statistical analysis using unpaired *t*‐test. Key: *p* < 0.05 = *, *p* < 0.01 = **, *p* < 0.001 = ***, *p* < 0.0001 = ****.

To further assess the observed differences in immunosuppressive features between mCAS‐A and mCAS‐B, we analysed the expression of various immune checkpoints, as summarised in Figure 6A, across mCAS subgroups. Strikingly, we observed a consistent significant reduction in the expression of inhibitory T‐cell receptors PDCD1, BTLA, HAVCR2 and CTLA4 (Figure 6B). While in contrast, the expression of most inhibitory ligands expressed on antigen‐presenting cells in mCAS‐B, namely CD274, TNFRSF14 and LGALS9 was significantly increased (Figure 6B). Additionally, we observed widespread decreased expression of stimulatory T‐cell receptors in mCAS‐B, significant for CD28, CD27 and TNFRSF4 (Figure 6C). Lastly, the expression of stimulatory ligands was significantly decreased in mCAS‐B, with the exception of CD86 (Figure 6C).

## 4 Discussion

Little is known about how the stromal reaction adapts in response to the degree of tumour malignancy observed in CCM and CMM. Although recent sequencing studies have contributed to improved understanding of canine cutaneous and mucosal melanoma tumour biology, the modulating influence of CAS on development and progression of these tumours has not been assessed until now [13, 14, 16, 36]. We present a comprehensive transcriptome‐wide analysis of stromal reprogramming in CCM and CMM and identify distinct immune expression profiles that may be associated with differences in observed tumour aggression. Our results indicate that CCM is coupled with limited stromal reprogramming, while CMM undergoes stronger deregulation, supporting the hypothesis that aggressive tumours may be supported by a more reactive stroma (Figures 2 and 3).

Initial characterisation of our CCM cohort using common prognostic factors revealed the majority of cases to have low MC, as expected. Interestingly, only the presence of tumour necrosis was associated with increased MC (Figure 1H). Necrosis was identified as a possible prognostic factor in canine cutaneous melanocytic neoplasms and has also been shown to apply to human cutaneous melanoma [2, 37].

We observed a high degree of overlap between cCAS and cNormal stroma following dimensionality reduction analysis (Figure 2A). From this, we expected limited stromal reprogramming in cCAS compared to cNormal, as confirmed in Figure 2B. CCM commonly presents as a less aggressive disease and as such may be linked to lower levels of stromal expression deregulation that are insufficient to support disease progression. Interestingly however, the expression of 19 DEGs significantly correlated with MC, suggesting that these genes may be involved in malignant development. Of these genes, VAV1, CD22 and CARD11 could be linked to B cell receptor signalling. Existing literature outlines a negative prognostic impact for CD20+ B cell tumour in canine melanoma [10]. Meanwhile, CARD11 has been identified as a prognostic factor in human uveal melanoma with an inverse relationship with monocyte and regulatory T‐cell tumour infiltration [38]. Furthermore, knockdown on VAV1 has been associated with a reduction in melanoma cell invasion, a key characteristic of aggressive disease [39]. Other immune‐related pathways were highlighted among the upregulated genes in cCAS compared to cNormal, linked to the expression of CD4 and CTLA4 (Figure 2D). CD4 is a key marker for T helper cells, while CTLA4 is a receptor found on T‐cells that functions as an inhibitory immune checkpoint.

In contrast to the limited stromal reprogramming in CCM, heightened stromal alterations observed in CMM may reflect a dynamic microenvironment conducive to tumour growth, relapse and metastasis, in accordance with recent literature in human follicular lymphoma and renal cell carcinoma [40, 41]. Our group also observed similar findings of enhanced reprogramming in canine mammary carcinoma compared to less malignant adenoma [20]. These findings underscore the importance of considering stromal characteristics to improve understanding of mechanisms of tumour progression.

Despite the detection of 3816 significant DEGs, refined correlation thresholds revealed only 14 genes to have strong correlation with MC. There was no overlap with these genes and correlation with MC in CCM, suggesting differential stromal manipulation by the corresponding tumours.

Heatmap presentation of all significant DEGs in mCAS compared to mNormal revealed two subgroups of mCAS. Interestingly, mCAS‐A presented with high expression of genes associated with developmental and neuronal pathways, which may be a driving feature of PC1 (Figure 3A). Meanwhile, consistent with all cases of mCAS was high expression of genes enriched among contractile and ECM‐related pathways. Fibroblast activation towards myofibroblast‐like state is a key characteristic of CAS and may support the aggressive progression of mucosal melanoma in dogs. However, no difference in key prognostic characteristics between mCAS‐A and mCAS‐B including tumour MC was detected. This may highlight that differences are driven by stromal expression rather than patterns in tumour characteristics.

Analysis of tumour immune infiltration in our cohorts of CCM and CMM identified a trend of increased MC in immune cell‐excluded tumours (Figures 2E and 3D) that remained consistent regardless of CMM. This suggests that while more aggressive tumours do trigger an immune response, they are able to detain the immune cells from entering the tumour tissue and exerting tumour control. Interestingly, we observed the lowest stromal expression levels of various immune‐associated targets in mCAS‐B. Furthermore, expression of all T‐cell immune checkpoint markers in the stroma was lower in mCAS‐B than mCAS‐A (Figure 6B). Interestingly, MC in the CD3 infiltrated samples was significantly higher in the mCAS‐B than mCAS‐A group, suggesting the lower immune reactivity to coincide with lower tumour control. Taken together, the low expression of immune‐associated genes and immune checkpoints may indicate that mCAS‐B CMM patients may respond poorly to immunotherapy [42]. However, further studies are necessary to correlate expression profiles of stromal immune cells with tumour‐infiltrating capabilities.

In conclusion, our study provides novel insights into the molecular and immunological features of canine melanoma, with a particular emphasis on the role of stromal reprogramming and immune dysregulation in mucosal subtypes.

## Author Contributions

E.M., A.M., and E.B. planned and initiated the study. E.B. performed LCM, RNA isolation and bioinformatic sequencing data processing and analysis. Z.M. and E.B. performed ISH and RT‐qPCR validation. A.M. and C.K. performed case selection and definition of regions of interest to be isolated. C.K. performed IHC. E.B., C.K., A.M., and E.M. performed data analysis. E.M. was responsible for study design, supervision and funding. E.B. and E.M. wrote the first draught of the manuscript. All authors read, contributed to and approved the final manuscript.

## Ethics Statement

According to the Swiss Animal Welfare Law Art. 3c, Abs. 4, the preparation of tissues in the context of agricultural production, diagnostic or curative operations on the animal or for determining the health status of animal populations is not considered an animal experiment and, thus, does not require an animal experimentation licence. All the FFPE specimens used were obtained for diagnostic reasons and did not therefore require a formal ethics approval, in full compliance with national guidelines.

## Conflicts of Interest

The authors declare no conflicts of interest.

## Supporting information


Figures S1–S5.

Tables S1–S6.

## Data Availability

The data that supports the findings of this study are available in the Supporting Information of this article.
