# Supplementary material for: Stromal Expression Profiling Reveals Immune‐Driven Adaption to Malignancy in Canine Melanoma Subtypes
Source: Vet Comp Oncol. 2024 Oct 17;23(1):20–9. doi: 10.1111/vco.13021 (PMC11830460; doi:10.1111/vco.13021)
Supplement: Supplementary file 1 — Figures S1–S5. [file VCO-23-20-s002.docx]

Supplementary Table 1. Overview of clinical cases used in the study.

Supplementary Table 2. Overview of primers used for validation by RT-qPCR.

| **Target** | **Reference** | **Amplicon length** |
| --- | --- | --- |
| GPC6 | Cf02704987_m1 | 84 nt |
| VIT | Cf02657286_m1 | 68 nt |
| ADAMTSL3 | Cf02643771_m1 | 59 nt |
| MYH2 | Cf02631729_g1 | 104 nt |
| TNN | Cf02678806_m1 | 83 nt |
| PPIA | Cf03986523_gH | 92 nt |
| B2M | Cf02659077_m1 | 87 nt |

Supplementary Table 3. Table of all differentially expressed genes in cCAS compared to cNormal.

Supplementary Table 4. Correlation co-efficient matrix of significant DEG expression with CCM mitotic count with cut-offs at r < 0.05 and r > 0.05.

Supplementary Table 5. Table of all differentially expressed genes in mCAS compared to mNormal.

Supplementary Table 6. Correlation co-efficient matrix of significant DEG expression with CMM mitotic count with cut-offs at r < 0.05 and r > 0.05.

**Supplementary Figure 1**. Graphical overview of workflow to analyse stromal expression in canine melanoma subtypes.

**Supplementary Figure 2**. Visual confirmation of microdissected tissue from stromal regions of interest. Scale bar not available.

**Supplementary Figure 3.** Volcano plot of significantly deregulated targets in oral mucosal CAS (mCAS) compared to mucocutaneous CAS (mcCAS) according to significant threshold values of padj < 0.05 and Log2FC > |1|.

**Supplementary Figure 4**. Characterisation of CMM subgroups. A) PCA plot of CMM with mCAS samples coloured by subgroup A or B. B) PCA plot of CMM with mCAS samples coloured by mucosal or mucocutaneous location. C) Mitotic count in mCAS-A compared to mCAS-B. Unpaired t-test, key: ns = not significant. D) GSEA of reactome pathways in mCAS-A compared to mNormal.

**Supplementary Figure 5.** Semi-quantitative analysis of tumour infiltrating immune cells using Visiopharm. Representative images of visiopharm heatmap (top row) with matched brightfield image of the same tumour region (bottom row). Left: CD20 by IHC (CCM01), middle: CD3 by in-situ hybridization (CMM10), right: CD20 by IHC (CCM8).
